# Collaborative representation based on enhanced tensor robust PCA for hyperspectral anomaly detection

**DOI:** 10.1371/journal.pone.0331894

**Published:** 2025-09-25

**Authors:** Ruhan A, Cheng Liang Zhong, Senjia Wang, Quanxue Gao

**Affiliations:** 1 Xianyang Normal University, Xianyang, Shaanxi, China; 2 Xi’an Peihua University, Xi’an, Shaanxi, China; 3 Tsinghua University, Beijing, China; 4 Zhengzhou University of Industrial Technology, Xinyang, Henan, China; 5 Xidian University, Xi’an, Shaanxi, China; University of Essex Faculty of Science and Engineering, UNITED KINGDOM OF GREAT BRITAIN AND NORTHERN IRELAND

## Abstract

This paper presents a novel hyperspectral anomaly detection (HAD) method, ETRPCA-CRD, which integrates enhanced tensor robust principal component analysis (ETRPCA) with collaborative representation detection (CRD) to effectively separate anomalous targets from background data. The key novelty lies in the use of weighted tensor Schatten-*p* norm minimization (WTSNM) within the ETRPCA framework, which assigns distinct weights to different singular values to preserve important information while eliminating noise. The ETRPCA problem is efficiently solved by Fourier transform, generalized soft-thresholding (GST), and T-singular value decomposition (SVD) methods. This approach significantly improves detection accuracy by fully utilizing the spectral-spatial information of hyperspectral images (HSIs) represented as tensors. The low-rank tensor obtained from ETRPCA serves as the background data for CRD, further enhancing detection performance. Experiments on three real hyperspectral datasets and one simulated dataset demonstrate that ETRPCA-CRD outperforms several state-of-the-art algorithms, achieving superior detection accuracy and robustness. The proposed method’s ability to effectively distinguish anomalies from background data while preserving salient signals makes it a powerful tool for hyperspectral anomaly detection.

## Introduction

In hyperspectral images (HSIs), accurately identifying anomalous targets can be challenging due to contamination by abnormal data and background noise. To address this problem, various algorithms have been developed to separate clean background data from anomalous target data, thereby improving detection performance.

Based on the principle that anomalous pixels cannot be linearly represented by their surrounding data while background pixels can, Li and Du presented a classic hyperspectral anomaly detection (HAD) method, known as collaborative representation detection (CRD) [1], in which background data are collected in a sliding dual window. CRD effectively detects anomalous pixels by removing the approximated background from the original HSIs. Building upon CRD, kernel collaborative representation detection was introduced to further improve the anomaly detection performance. To address the problem of potential outliers in the sliding dual window in CRD, Su et al. proposed CRD with Principal Component Analysis (PCA) remove outlier (PCAroCRD) [2], which uses principal component analysis to purify the background data. In this approach, the top principal components contain the most background information, while the latter contain anomaly-related information. This purification process improves the anomaly detection accuracy. Yin et al. proposed a method called the selective search collaborative representation detector for HAD [3]. Their method utilizes global information and spectral similarity to fuse similar pixels. This selective search technique purifies the local background estimation and reduces the reliance on the dual window size in CRD. Another approach, known as the copula-based CRD, was introduced to address challenges related to limited spatial information use and anomalous target contamination [4]. In this approach, background purification is performed using copula-based outlier detection, and spatial difference enhancement is achieved using guided filtering, effectively distinguishing anomalous targets from the background. Additionally, Ma et al. proposed a novel representation-theory-based CRD approach that allows the detector to use nonlinear features beyond the Gaussian distribution [5]. Furthermore, they proposed an anomaly contamination detection system to address potential false positives due to neighboring anomalous pixels. Hu et al. introduced an algorithm for target detection based on binary-class collaborative representation (BCRD) [6], where background data are linearly represented by surrounding pixels, and anomalous targets are approximated by prior information. Anomaly detection is achieved by estimating the residuals of both representations. BCRD enhances the detection accuracy through background dictionary purification; however, it requires prior knowledge of anomalous targets, which is often challenging in practical HAD applications.

Additionally, low-rank and sparse matrix decomposition-based methods can precisely distinguish between abnormal target and background data. The low-rank and sparse matrix decomposition detector (LRaSMD) [7] was developed based on the assumption that noise can be simulated by Gaussian random variables, that abnormal targets follow a sparse distribution, and that the background image is low-rank. In addition, the low-rank and sparse matrix decomposition-based Mahalanobis distance (LSMAD) [8] suppresses abnormal target contamination in the background.

Robust principal component analysis(RPCA) [9] decomposes a matrix into low-rank and sparse components. However, it may not effectively approximate rank in practice. Additionally, nuclear norm regularization in RPCA shrinks all singular values equally, although different singular values contain different information. To address this limitation, methods such as partial sum minimization of singular values in Robust PCA(PSSV) [10], truncated nuclear norm [11], and weighted nuclear norm minimization [12] have been proposed to preserve important information.

On the basis of the above research, we propose the enhanced tensor RPCA-CRD (ETRPCA-CRD) method for HAD, in which tensor data are reconstructed into a low-rank tensor and sparse tensor using ETRPCA. Weighted tensor Schatten-*p* norm minimization (WTSNM) retains important information by treating different singular values differently, and an efficient method is employed to solve ETRPCA. In addition, the use of 𝒳 as the background data for CRD effectively enhances the detection performance and accuracy. Our proposed method is compared with several state-of-the-art methods on three real datasets and one simulated hyperspectral dataset.

- In HAD, there are both spectral and spatial differences between abnormal targets and surrounding background data. By ignoring the spatial correlation of the data, previous HAD approaches expanded three-dimensional (3D) hyperspectral data into two-dimensional matrices. In this paper, HSIs are represented by tensors to retain the original information and structure of the data and fully utilize the spectral and spatial information of the HSIs in detection.
- The tensor data are reconstructed into a low-rank tensor 𝒳 and sparse tensor ℰ using the ETRPCA method. The background information in 𝒳 is retained, whereas anomalous targets and noisy information are removed. Therefore, using 𝒳 as the background data for the CRD algorithm effectively enhances the detection performance and accuracy.
- Our proposed method employs WTSNM, in which each singular value is treated separately. Therefore, the important information of large singular values is retained due to less shrinkage. By assigning a specific weight to each singular value, the salient signals and filters can be preserved out of noise, greatly improving the detection accuracy.
- The ETRPCA problem is efficiently solved by Fourier transform, generalized soft-thresholding (GST), and T-singular value decomposition (SVD) methods.

## Proposed algorithm

Tensor decomposition has been selected as the core technique for processing hyperspectral data in this study, owing to its unique advantages in handling the high dimensionality and complex structure of such data. By representing hyperspectral images as multi-dimensional tensors, tensor decomposition preserves their inherent spectral-spatial structure, thereby avoiding the information loss often associated with traditional matrix flattening approaches. This method is particularly effective in capturing the multilinear relationships between different dimensions, which is crucial for accurately identifying anomalies that exhibit distinct spectral signatures and spatial patterns. The enhanced tensor robust principal component analysis (ETRPCA) employed in this study is designed to be robust against noise and outliers, enabling more reliable separation of background and anomaly signals. The computational complexity associated with ETRPCA is effectively managed through the use of Fourier transforms, generalized soft-thresholding (GST), and T-singular value decomposition (SVD) methods. These techniques collectively ensure that the ETRPCA problem can be solved efficiently, thereby addressing the inherent computational challenges and maintaining the practicality of the method for large-scale datasets.

This paper adopts a notation convention for different mathematical entities. Matrices (e.g., 𝐀), vectors (e.g., 𝐚), and scalars (e.g., *a*) are represented by uppercase bold letters, lowercase bold letters, and lowercase letters, respectively. An n×n identity matrix is represented by $I_{n}$, while a third-order tensor is denoted by bold calligraphic letters (e.g., $𝒜\in {\mathbb{R}}^{n_{1}\times n_{2}\times n_{3}}$). Real and complex fields are denoted by ℝ and ℂ, respectively. The entries of a tensor 𝒜 are denoted by ${𝒜}_{ijk}$ or simply *a*_*ijk*_, where $𝒜\in {\mathbb{R}}^{n_{1}\times n_{2}\times n_{3}}$. Slices of 𝒜 are represented as 𝒜(i,:,:) (horizontal slice), 𝒜(:,i,:) (lateral slice), and 𝒜(:,:,i) (frontal slice). A matrix representing the *i*-th frontal slice is denoted by ${𝐀}^{\left(i\right)}=𝒜(:,:,i)$. A tube of a third-order tensor is defined by fixing the first two indices and varying the third, such as 𝒜(i,j,:), representing the *ij*-th tube of 𝒜. The inner product between matrices 𝐀 and 𝐁 in ${\mathbb{C}}^{n_{1}\times n_{2}}$ is defined as $\langle 𝐀,𝐁\rangle =\mathrm{tr}({𝐀}^{*}𝐁)$, where ${𝐀}^{*}$ denotes the conjugate transpose. For tensors 𝒜 and ℬ in ${\mathbb{C}}^{n_{1}\times n_{2}\times n_{3}}$, their inner product is defined as $\langle 𝒜,ℬ\rangle =\sum_{i=1}^{n_{3}}\left\langle A^{\left(i\right)},B^{\left(i\right)}\right\rangle$. In the context of real tensors $𝒜\in {\mathbb{R}}^{n_{1}\times n_{2}\times n_{3}}$, the result of the discrete fast Fourier transform (FFT) along the third dimension is represented by $\overset{―}{𝒜}\in {\mathbb{C}}^{n_{1}\times n_{2}\times n_{3}}$, denoted by FFT(𝒜,[],3). The inverse FFT is denoted by $\mathrm{IFFT}(\overset{―}{𝒜},[],3)$. $\overset{―}{𝒜}$ can be expressed as a block diagonal matrix, where each block on the diagonal is the frontal slice ${\overset{―}{A}}^{\left(i\right)}$ of $\overset{―}{𝒜}$[13]. Several norms of matrix **A** employed in this paper are the $\ell_{1}$-norm $\|𝒜{\|}_{1}=\sum_{ijk}\left|a_{ijk}\right|$, Frobenius norm $\|𝒜{\|}_{F}=\sqrt{\sum_{ijk}{\left|a_{ijk}\right|}^{2}}$, nuclear norm $\left\|𝐀{\|}_{*}=\sum_{i}\sigma_{i}(𝐀\right)$, and $\ell_{2}$-norm $\|𝐀{\|}_{2}=\sqrt{\sum_{ij}{\left|{𝐚}_{ij}\right|}^{2}}$. The where X is a low-rank tensor (Background information) and E is a sparse tensor (noise and anomalous target). To accurately recover low-rank tensors and sparse tensors, in previous studies, tensor nuclear norm minimization[14] was used as a convex relaxation. However, in solving the tensor nuclear norm minimization problem, all singular values are regularized and shrinked equally. Different singular values contain different information, especially the first several large singular values, which contain most of the prominent information. To preserve the prominent information, first several large singular values should shrink less. Therefore, the weighted tensor Schatten-*p* norm [15] is used in this paper.

To preserve the original spectral and spatial information of the HSIs, we represent the hyperspectral data by a 3D tensor, denoted by $𝒴\in {\mathbb{R}}^{n_{1}\times n_{2}\times n_{3}}$. We then decompose this tensor, 𝒴, into a low-rank tensor 𝒳 and sparse tensor ℰ:

𝒴=𝒳+ℰ, (1)

where 𝒳 represents background information, and ℰ contains noise and anomalous targets. Tensor nuclear norm minimization (TNNM) was previously employed as a convex relaxation for recovering low-rank and sparse tensors. However, TNNM tends to regularize and shrink all singular values equally. Since different singular values contain different information, it is crucial to shrink the first several large singular values less because they contain most of the important information. Therefore, this paper adopts the weighted tensor Schatten-*p* norm (WTSN) [15–17]. For $𝒳\in {\mathbb{R}}^{n_{1}\times n_{2}\times n_{3}}$ and $h=\min(n_{1},n_{2})$, the WTSN of 𝒳 is defined as

$$\|X{\|}_{\omega ,S_{p}}={\left(\sum_{i=1}^{n_{3}}{\left\|{\overset{―}{𝐗}}^{\left(i\right)}\right\|}_{\omega ,S_{p}}^{p}\right)}^{\frac{1}{p}}\;={\left(\sum_{i=1}^{n_{3}}\sum_{j=1}^{h}\omega_{j}*\sigma_{j}{\left({\overset{―}{𝐗}}^{\left(i\right)}\right)}^{p}\right)}^{\frac{1}{p}},$$ (2)

where $\omega =[\omega_{1},\ldots ,\omega_{h}]$ denotes a nonnegative weighted vector. The weights are set to the inverse ratio of the singular values ${𝐘}^{\left(i\right)}$ to ensure less shrinkage of the first several large singular values: $w_{i}=C\sqrt{n_{1}n_{2}}/\left(\sigma_{i}\left({𝐘}^{\left(i\right)}\right)+\varepsilon \right)$, where *C* is a compromising constant, $\sigma_{j}(•)$ denotes the *j*-th largest singular value of a matrix, *ε* is a small positive number to avoid division by zero, and *p* (0 < *p* < 1) is the power of the matrix singular value.

The objective function is given by

$$\min_{E,X}\lambda \|E{\|}_{1}+\|X{\|}_{\omega ,S_{p}}^{p}\;\text{ s.t. }𝒴=X+E$$ (3)

where ℒ is the Lagrange Multiplier and *μ* is a positive scaler. From this problem, update ℰ with other fixed variables is given by By solving Eq 3, we can recover X and E. The optimization problem is solved using the augmented Lagrange multiplier method. The constrained problem is converted into an unconstrained one with additional penalty terms, as follows:

$$\Gamma (ℰ,𝒳,ℒ,\mu )=\lambda {\left\|ℰ\right\|}_{1}+\langle ℒ,𝒴-𝒳-ℰ\rangle \;\|𝒳{\|}_{\omega ,S_{p}}^{p}+\frac{\mu }{2}\|𝒴-𝒳-ℰ{\|}_{F}^{2}$$ (4)

Here, *μ* is a positive scalar, and ℒ is the Lagrange multiplier. Letting other variables be fixed, ℰ is updated as follows:

$${ℰ}_{k+1}=\underset{ℰ}{\arg\min}\frac{\lambda }{\mu_{k}}\|ℰ{\|}_{1}+\frac{1}{2}{\left\|ℰ-{ℋ}_{k}\right\|}_{F}^{2}$$ (5)

Here, ${ℋ}_{k}=𝒴+\mu_{k}^{-1}{ℒ}_{k}-{𝒳}_{k}$. Inspired by the soft-thresholding operator, we obtain

$${ℰ}_{k+1}=T_{\frac{\lambda }{\mu_{k}}}\left({ℋ}_{k}\right).$$ (6)

Here, the (*i*,*j*,*k*)-th element of $T_{\frac{\lambda }{\mu_{k}}}\left({ℋ}_{k}\right)$ is $\mathrm{sign}\left({\left({ℋ}_{k}\right)}_{i,j,k}\right)•\max\left(\left|{\left({ℋ}_{k}\right)}_{i,j,k}\right|-\frac{\lambda }{\mu_{k}},0\right)$.

With other variables fixed, 𝒳 is updated as follows:

$${𝒳}_{k+1}=\underset{𝒳}{\arg\min}\mu_{k}^{-1}\|𝒳{\|}_{\omega ,S_{p}}^{p}+\frac{1}{2}{\left\|𝒳-{ℳ}_{k}\right\|}_{F}^{2}$$ (7)

Here, ${ℳ}_{k}=𝒴+\mu_{k}^{-1}{ℒ}_{k}-{ℰ}_{k+1}$, which is referred to as WTSNM [15–17] and can be efficiently solved using GST.

The other variables are updated as follows:

$${ℒ}_{k+1}={ℒ}_{k}+\mu_{k}\left(𝒴-{𝒳}_{k+1}-{ℰ}_{k+1}\right)$$ (8)

$$\mu_{k+1}=\min\left(\rho \mu_{k},\mu_{\max}\right)$$ (9)

GST is employed to effectively solve the WTSNM problem, as briefly described below.

**Theorem 1.** *For m×n matrices* **A** *and* **B***, with singular values denoted by $\sigma (𝐀)={\left[\sigma_{1}(𝐀),\ldots ,\sigma_{r}(𝐀)\right]}^{T}$ and $\sigma (𝐁)={\left[\sigma_{1}(𝐁),\ldots ,\sigma_{r}(𝐁)\right]}^{T}$, where r=min(m,n), it holds that $\mathrm{tr}({𝐀}^{T}𝐁)\leq \mathrm{tr}(\sigma {\left(𝐀\right)}^{T}\sigma (𝐁))$. Equality occurs if and only if there exist unitaries* **U** *and* **V** *such that $𝐀=𝐔\Sigma_{A}{𝐕}^{T}$ and $𝐁=𝐔\Sigma_{B}{𝐕}^{T}$, where $\Sigma_{A}$ and $\Sigma_{B}$ are ordered eigenvalue matrices with singular values σ(𝐀) and σ(𝐁) along the diagonal with the same order, respectively [18,19].*

**Lemma 1.** *Let the SVD of* **Y** *be $𝐘=𝐔\Sigma {𝐕}^{T}$ with $\Sigma =\mathrm{diag}(\sigma_{1},\ldots ,\sigma_{r})$, assuming a nonascending order of all singular values [18,19]. For a given τ>0, l=min(m,n), and $0\leq \omega_{1}\leq \omega_{2}\leq \ldots \leq \omega_{l}$, the following model,*

$$\underset{𝐗}{\arg\min}\frac{1}{2}\|𝐗-𝐘{\|}_{F}^{2}+\tau \|𝐗{\|}_{\omega ,S_{p}}^{p},$$ (10)

*has an optimal solution $𝐗=𝐔{𝐏}_{\tau *\omega }(𝐘){𝐕}^{T}$ with ${𝐏}_{\tau *\omega }(𝐘)=\mathrm{diag}(\delta_{1},\ldots ,\delta_{r})$. Here, by solving r independent subproblems, we can obtain $\delta_{i}$, where*

$$\min_{\delta_{i}\geq 0}f(\delta_{i})={\left(\delta_{i}-\sigma_{i}\right)}^{2}+\omega_{i}\delta_{i}^{p},i=1,\ldots ,r.$$ (11)

The subproblems in Eq 11 can be effectively solved using GST. Given *p* and *δ*, a specific threshold exists:

$$\tau_{p}^{GST}\left(\omega_{i}\right)={\left(2\omega_{i}(1-p)\right)}^{\frac{1}{2-p}}+\omega_{i}p{\left(2\omega_{i}(1-p)\right)}^{\frac{p-1}{2-p}}$$ (12)

For the subproblem defined in Eq 11, the optimal solution depends on the relationship between $\sigma_{i}$ and the threshold $\tau_{p}^{GST}(\omega_{i})$. If $\sigma_{i}<\tau_{p}^{GST}(\omega_{i})$, then the optimal solution of Eq 11 is 0. In contrast, if $\sigma_{i}>\tau_{p}^{GST}(\omega_{i})$, the optimal solution of Eq 11 is $\mathrm{sign}(\sigma )S_{p}^{GST}\left(\sigma_{i};\omega_{i}\right)$ and involves solving the following equation:

$$S_{p}^{GST}(\sigma_{i},\omega_{i})-\sigma_{i}+\omega_{i}p{\left(S_{p}^{GST}(\sigma_{i},\omega_{i})\right)}^{p-1}=0$$ (13)

This condition captures a soft-thresholding operation on the singular values. If the singular value is above the threshold, the soft-thresholding function $S_{p}^{GST}(\sigma_{i},\omega_{i})$ is applied, and the solution is obtained by solving the indicated equation. This process facilitates the efficient handling of cases where the singular values should be thresholded to achieve the optimal solution for the given problem. where ${\overset{―}{𝐗}}^{\left(i\right)}$ is the *i*-frontal slice of $\overset{―}{𝒳}$, ${\overset{―}{𝐌}}^{\left(i\right)}$ is the *i*-frontal slice of $\overset{―}{ℳ}$. Eq 14 can be decomposed into *n*_3_ ($i=1,2,\ldots ,n_{3}$) independent subproblems, and the *i*-th subproblem is as follows:

As expressed in Eq 7, the WTSNM problem is first transformed into the Fourier domain. The transformed problem can be represented as follows:

$$\underset{\overset{―}{𝒳}}{\mathrm{argmin}}\sum_{i=1}^{n_{3}}\left(\frac{1}{2}{\left\|{\overset{―}{𝐗}}^{\left(i\right)}-{\overset{―}{M}}_{k}^{\left(i\right)}\right\|}_{F}^{2}+\tau *{\left\|{\overset{―}{𝐗}}^{\left(i\right)}\right\|}_{\omega ,S_{p}}^{p}\right),$$ (14)

where ${\overset{―}{𝐗}}^{\left(i\right)}$ and ${\overset{―}{𝐌}}^{\left(i\right)}$ represent the *i*-th frontal slice of $\overset{―}{𝒳}$ and $\overset{―}{ℳ}$, respectively ($i=1,2,\ldots ,n_{3}$).

Eq 14 can be decomposed into *n*_3_ independent subproblems using the following formulation:

$$\underset{{\overset{―}{𝒳}}^{\left(i\right)}}{\mathrm{argmin}}\frac{1}{2}{\left\|{\overset{―}{𝐗}}^{\left(i\right)}-{\overset{―}{M}}_{k}^{\left(i\right)}\right\|}_{F}^{2}+\tau *{\left\|{\overset{―}{𝐗}}^{\left(i\right)}\right\|}_{\omega ,S_{p}}^{p},$$ (15)

where $i=1,2,\ldots ,n_{3}$. This decomposition enables the independent solution of each subproblem, simplifying the overall optimization process.

According to Lemma 1, ${𝐏}_{\tau *\omega }\left({\overset{―}{𝐌}}_{k}^{\left(i\right)}\right)=\mathrm{diag}\left(\delta_{1},\ldots ,\delta_{r}\right)$, $\delta_{i}=T_{p}^{GST}\left(\sigma_{i}({\overset{―}{𝐌}}_{k}^{\left(i\right)}),\tau *\omega_{i}\right)$, the optimal solution of Eq 15 is ${\overset{―}{𝐗}}^{(i)*}={\overset{―}{𝐔}}^{\left(i\right)}{𝐏}_{\tau *\omega }\left({\overset{―}{M}}_{k}^{\left(i\right)}\right){\overset{―}{𝐕}}^{(i)T}$, where ${\overset{―}{𝐗}}^{(i)*}$, ${\overset{―}{𝐔}}^{(i)*}$, and ${\overset{―}{𝐕}}^{(i)*}$ denote the *i*-th frontal slice of ${\overset{―}{𝒳}}^{*}$, ${\overset{―}{𝒰}}^{*}$, and ${\overset{―}{𝒱}}^{*}$, respectively. By traversing 1 to *n*_3_, we obtain the optimal solution of each subproblem. Then, according to T-SVD [13], we obtain the optimal solution of Eq 7 (WTSNM).

$$X^{*}=𝒰*IFFT\left({𝐏}_{\tau *\omega }({\overset{―}{M}}_{k})\right)*{𝒱}^{T},$$ (16)

where $𝒰=IFFT(\overset{―}{𝒰},[],3)$ and $𝒱=IFFT(\overset{―}{𝒱},[],3)$

Solving the optimization problem Eq 4 using this approach yields a low-rank tensor 𝒳 and sparse tensor ℰ. The former contains the global background data, while the latter contains anomaly data. The separation of anomalies from background is thereby achieved. Therefore, 𝒳 can be applied to the CRD algorithm to achieve background purification and improve detection performance.

${𝐲}_{i}=[y_{i1},y_{i2},\ldots ,y_{ij}]\in {\mathbb{R}}^{n_{3}\times 1}$ is the spectral vector of a pixel in $Y\in {\mathbb{R}}^{n_{1}\times n_{2}\times n_{3}}$. 𝒳 serves as clean background data by eliminating noise and anomalous targets, making it suitable for collecting background data in the CRD algorithm. By using a local dual-window approach, we collect background data from 𝒳, resulting in a set of selected samples denoted by ${𝐗}_{s}={\left\{{𝐱}_{i}\right\}}_{i=1}^{s}$, where $s=w_{\text{out }}\times w_{\text{out }}-w_{\text{in }}\times w_{\text{in }}$ and *s* represents the number of selected samples. The CRD algorithm formula is as follows:

$$\arg\min_{\alpha }{\left\|𝐲-{𝐗}_{s}\alpha \right\|}_{2}^{2}+\lambda {\left\|\Gamma_{𝐲}\alpha \right\|}_{2}^{2},$$ (17)

and its solution is

$$\hat{\alpha }={\left({𝐗}_{s}^{T}{𝐗}_{s}+\lambda \Gamma_{𝐲}^{T}\Gamma_{𝐲}\right)}^{-1}{𝐗}_{s}^{T}𝐲,$$ (18)

where $\Gamma_{y}$ is

$$\Gamma_{𝐲}=\left[\begin{array}{ccc} {\left\|𝐲-{𝐱}_{1}\right\|}_{2} & & 0\\ & \ddots & \\ 0 & & {\left\|𝐲-{𝐱}_{s}\right\|}_{2} \end{array}\right].$$ (19)

In CRD, anomalies can be determined from the following residual image:

$$r_{1}=\|𝐲-\hat{𝐲}{\|}_{2}={\left\|𝐲-{𝐗}_{s}\hat{\alpha }\right\|}_{2}$$ (20)

Here, *y* is an anomalous pixel if *r*_1_ is above the threshold. We refer to this HAD method as ETRPCA-CRD.

## Experiments

### Datasets

The detection performance of ETRPCA-CRD was experimentally evaluated on three real hyperspectral datasets and one simulated dataset. All experiments were conducted on a system featuring an E5-2680v3 CPU, 32 GB of DDR4 memory, a 1-TB NVMe SSD, and an RTX 3060 graphics card with 12 GB of VRAM [20].

- AVIRIS-I: The San Diego Naval Airport HSI, originally taken by the AVIRIS sensor, has an image size of 400×400 pixels, a spatial resolution of 3.5 m per pixel, and 224 spectral bands covering the wavelength range of 370–2510 nm. We eliminated low-SNR, sensor fault, and water vapor absorption bands (1–6, 33–35, 97, 107–113, 153–166, 221–224), obtaining an image with 189 spectral bands. Finally, we prepared the AVIRIS-I dataset by capturing a 120×120 subimage from the top left corner of the obtained image, containing numerous ground objects and exhibiting a complex distribution. Three airplanes of 58 pixels were used as anomalies for experimental purposes.
- AVIRIS-II: A 100×100 pixel dataset was selected from the central area of the San Diego Naval Airport image, containing various types of ground objects. Three airplanes of 134 pixels were selected as anomalous targets in the experiment. The target morphology was more complete, and the spectral difference between the target and background was distinct.
- abu-urban-3: This dataset was derived from the urban scene of the ABU dataset [21] taken at Gainesville by the AVIRIS sensor. The image has a size of 100 × 100 pixels, a spatial resolution of 3.5 m per pixel, and 191 spectral bands. Various vehicles and other artificial targets were selected as anomalous targets.
- Salinas-simulated: This simulated dataset was generated by embedding anomalous targets in the real Salinas dataset. The anomalies were six square regions duplicated in reverse order and arranged in another line at a close distance, and their spectral bands were generated using a linear mixing model.

### Evaluation indicators

In this study, a color detection map (CDM) was employed as an intuitive indicator to qualitatively evaluate the detection performance of different HAD algorithms. The commonly used ROC curve and area under the curve (AUC) were selected as accuracy measures to compare the HAD algorithms. The more the ROC curve hugs the top left corner, the closer the AUC value is to 1, indicating a superior HAD algorithm [20].

### Detection results

We compared ETRPCA-CRD with CRD [1], LRaSMD [7], LSMAD [8], RPCA [9], SSRX [22], TenB [23], and MFIFD [24]. In ETRPCA-CRD, the regularization parameter *λ* equals $\frac{1}{\sqrt{max(n_{1},n_{2})n_{3}}}$, and *μ* is a positive scalar. The compromising constant is *C* = 10^1/*p*^ when solving the WTSNM problem. The *P* value ranges from 0.5 to 0.95 with an interval of 0.5, and the optimal *P* is determined when achieving the maximum AUC in the experiment. *ρ* is set to 1.2 when updating *μ* [13]. The optimal window size for CRD was determined via grid search with ranges from 11×13 to 33×35, by maximizing the AUC value. Table 1 presents the AUC values of ETRPCA-CRD and the seven other algorithms.

**Table 1:** AUC value comparison.

dataset	CRD	LRaSMD	LSMAD	RPCA	TenB	SSRX	MFIFD	ETRPCA-CRD
AVIRIS-I	0.9925	0.7879	0.9713	0.9110	0.9160	0.9852	0.9595	**0.9990**
AVIRIS-II	0.9671	0.8668	0.9670	0.9402	0.9326	0.9742	0.9597	**0.9846**
abu-urban-3	0.9716	0.9474	0.9661	0.9511	0.9468	0.9525	0.9541	**0.9979**
Salinas-simulated	0.7089	0.9991	0.9980	0.9861	0.9144	0.9975	0.9993	**0.9999**

**Fig 1:**
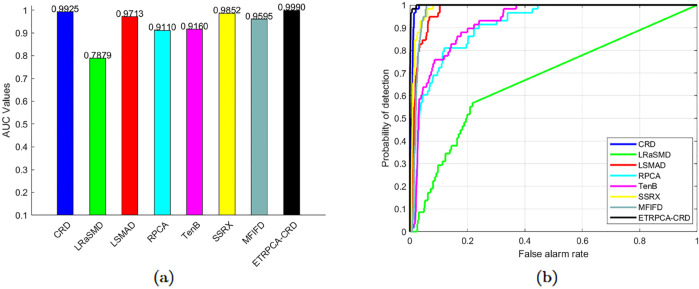
(a) AUC values and (b) ROC curves for the AVIRIS-I dataset.

**Fig 2:**
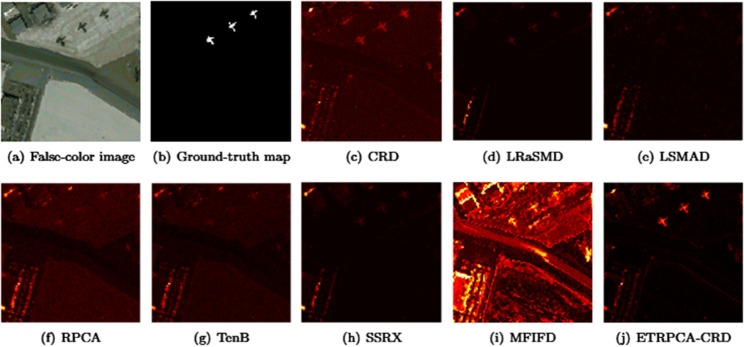
Color detection maps for AVIRIS-I dataset.

On the AVIRIS-I dataset, the optimal window size for CRD is determined to be (25, 23). The principal component numbers (K1, K2, and K3) in TenB are all set to 1. ETRPCA-CRD yields the best result at *p* = 0.2. Table 1 and Fig 1 present the AUC values. ETRPCA-CRD exhibits a significantly higher AUC value compared to that of the other algorithms, and the ranking of algorithms in terms of decreasing AUC is as follows: ETRPCA-CRD, CRD, SSRX, LSMAD, MFIFD, TenB, RPCA, and LRaSMD. Fig 1 presents the ROC curves of the eight algorithms, revealing that the ROC curves of the other algorithms are below that of ETRPCA-CRD. Fig 2 presents the CDMs of the compared methods, indicating that LRaSMD fails to detect anomalies in the experiment. The positions of the three airplanes are detected by LSMAD, RPCA, TenB, and SSRX, with blurred airplane shapes. CRD, RPCA, MFIFD, and TenB do not effectively separate anomalous and background pixels, resulting in poor anomaly detection. LSMAD and SSRX effectively suppress background pixels; however, they display dim abnormal targets. Compared with the other algorithms, ETRPCA-CRD accurately reconstructs the positions and shapes of the three airplanes and effectively distinguishes them from the background, exhibiting superior detection performance.

**Fig 3:**
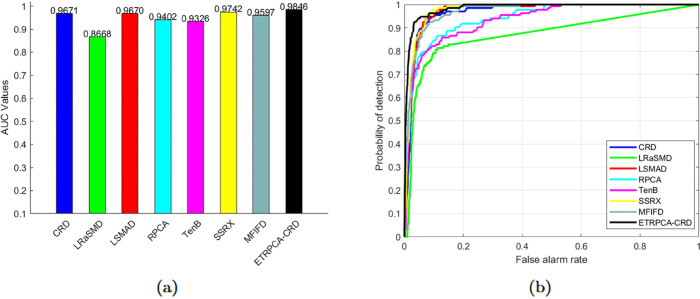
(a) AUC values and (b) ROC curves for the AVIRIS-II dataset.

**Fig 4:**
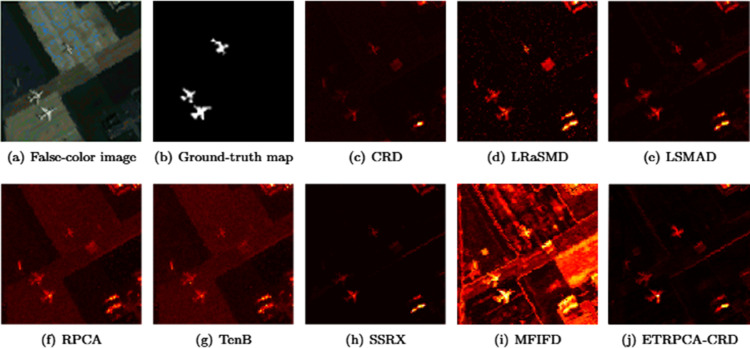
Color detection maps for the AVIRIS-II dataset.

On the AVIRIS-II dataset, the optimal window size for CRD is determined to be (19, 17). K1, K2, and K3 in TenB are all set to 3. ETRPCA-CRD achieves the optimal result at *p* = 0.15. Fig 3 presents the ROC curves of the eight algorithms. Of all ROC curves, that of ETRPCA-CRD is closest to the top left corner. Fig 3 and Table 1 present the AUC values. The ranking of the algorithms in terms of decreasing AUC is as follows: ETRPCA-CRD, SSRX, CRD, LSMAD, MFIFD, RPCA, TenB, and LRaSMD. In comparison with the other methods, ETRPCA-CRD provides a significantly higher AUC value of 0.9834. This is because each singular value is assigned an individual weight in ETRPCA-CRD, which effectively preserves salient signals and eliminates noise. Fig 4 presents the CDMs of the different methods. It can be seen that MFIFD, RPCA, and TenB perform poorly in separating anomalous and background pixels. They correctly detect the positions of the three airplanes; however, the airplane shapes are unclear. LRaSMD and LSMAD accurately detect the positions of the three airplanes; however, one of them has an incomplete shape. CRD and SSRX perform well in background suppression; however, there are two airplanes with incomplete shapes. In contrast, ETRPCA-CRD determines the accurate positions and complete shapes of all three airplanes, thereby achieving the best performance.

**Fig 5:**
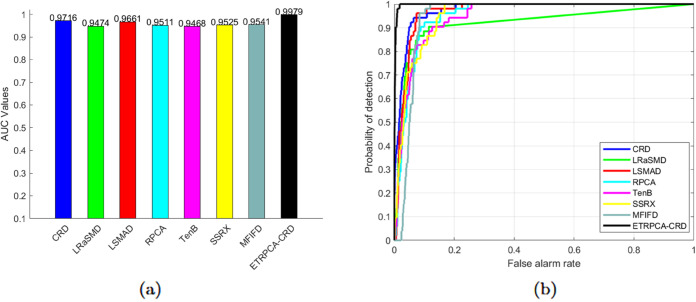
(a) AUC values and (b) ROC curves for the abu-urban-3 dataset.

**Fig 6:**
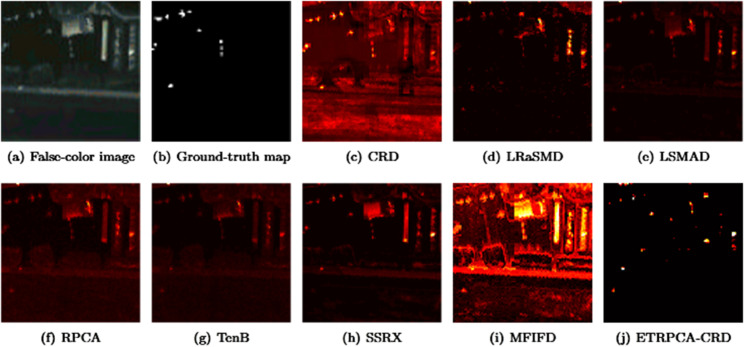
Color detection maps for abu-urban-3 dataset.

On the abu-urban-3 dataset, the optimal window size for CRD is determined to be (23, 21). K1, K2, and K3 in TenB are set to 4, 1, and 1, respectively. ETRPCA-CRD achieves the optimal result at *p* = 0.15. As illustrated in Table 1 and Fig 5, ETRPCA-CRD, which has an AUC value of 0.9899, outperforms the other algorithms. The algorithms are ranked in terms of decreasing AUC as follows: ETRPCA-CRD, CRD, LSMAD, MFIFD, SSRX, RPCA, TenB, and LRaSMD. Fig 5 presents the ROC curves of the eight algorithms. Of all ROC curves, that of ETRPCA-CRD is closest to the top left corner. Fig 6 presents the CDMs of the different algorithms. It can be seen that LRaSMD, LSMAD, RPCA, TenB, and SSRX mistakenly classify numerous background pixels as anomalous, while CRD and MFIFD fail to effectively separate the background from the anomalous targets. Overall, the proposed algorithm exhibits effective background suppression, achieving the best detection performance among the compared algorithms.

**Fig 7:**
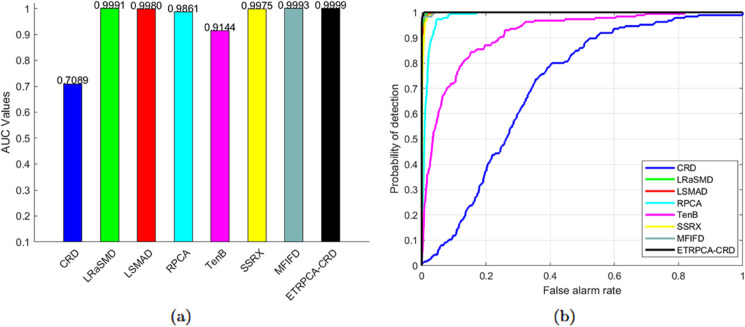
(a) AUC values and (b) ROC curves for the Salinas-simulated dataset.

**Fig 8:**
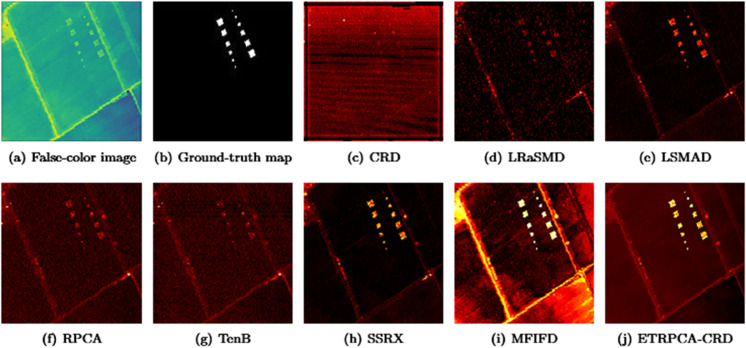
Color detection maps for Salinas-simulated dataset.

On the Salinas-simulated dataset, the optimal window size for CRD is determined to be (19, 17). K1, K2, and K3 in TenB are all set to 3. ETRPCA-CRD achieves the best results at *p* = 0.15. As illustrated in Table 1 and Fig 7, ETRPCA-CRD surpasses the other algorithms in terms of its AUC value of 0.9999, which is almost 1. The ranking of the algorithms in terms of decreasing AUC is as follows: ETRPCA-CRD, MFIFD, LRaSMD, LSMAD, SSRX, RPCA, TenB, and CRD. Fig 7 presents the ROC curves of the eight algorithms. The ROC curve of ETRPCA-CRD overlaps with the top left corner, while the curves of the other algorithms are below it. Fig 8 displays the CDMs of the different algorithms. It can be seen that CRD fails to detect anomalies. LRaSMD, LSMAD, RPCA, TenB, MFIFD, and SSRX can detect the positions and shapes of all abnormal targets; however, they perform poorly in separating anomalous pixels from background pixels. In contrast, ETRPCA-CRD successfully detects the positions and shapes of all anomalous targets, which appear prominently bright.

**Fig 9:**
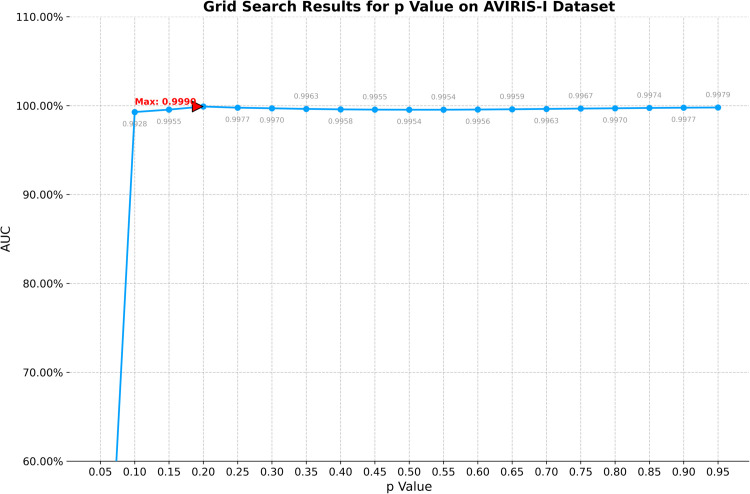
Grid search results for p value on AVIRIS-I dataset.

## Parameter analysis

1) **Analysis of *p***: The efficacy of the proposed method exhibits significant dependence on two critical hyperparameters: the dual-window configuration in contextual region detection (CRD) and the Schatten *p*-norm coefficient in weighted tensor Schatten *p*-norm minimization (WTSNM). To determine optimal parameterization, a systematic grid search protocol was executed. The *p*-value space spanning [0.05, 0.95] was discretized into 19 equidistant points (step size 0.05). The parameter analysis for the *p* value across four datasets is shown in Figs 9 through 12, revealing distinct trends in model performance as measured by AUC.
The AVIRIS-I dataset presents the most stable performance profile. After reaching a maximum AUC of 0.9990 at *p* = 0.15, the model maintains AUC values above 0.9950 across nearly the entire p value spectrum from 0.10 to 0.95, with only minimal variation observed.
  On the AVIRIS-II dataset, the AUC exhibits a near-linear increase from 0.7026 at *p* = 0.05 to a peak of 0.9846 at *p* = 0.10. Beyond this point, the AUC stabilizes, maintaining values above 0.9820 across *p* values from 0.15 to 0.95, indicating robust performance across this range. For the abu-urban-3 dataset, the AUC rises dramatically from 0.7663 at *p* = 0.05 to 0.9979 at *p* = 0.15. Performance then exhibits slight fluctuation around 0.9850 between *p* = 0.20 and *p* = 0.95, with minor deviations of ±0.0050 from this mean value.
  The Salinas-simulated dataset demonstrates a similar pattern but with higher absolute AUC values. Here, the maximum AUC of 0.99993 is achieved at *p* = 0.15. However, unlike AVIRIS-II, this dataset shows a plateau of near-maximal performance spanning *p* = 0.10 to *p* = 0.25, with AUC values exceeding 0.9950 in this interval.
  Collectively, these results indicate that optimal p values typically fall between 0.10 and 0.20 across datasets, with performance plateaus suggesting flexibility in parameter selection while maintaining high predictive accuracy. The parameter tuning process confirms the importance of dataset-specific optimization within this critical p value range.
2) **Analysis of dual-window size**: For the dual-window size, configurations ranging from 11×13 to 33×35 were tested, with the configuration yielding the highest AUC selected as optimal. The grid search results are presented in Tables 2, 3, 4, and 5.
Table 2 illustrates the grid search results for the AVIRIS-I dataset. The optimal dual-window configuration is identified as 23×25, achieving an AUC of 0.9990. For the AVIRIS-II dataset (Table 3), the 17×19 configuration provides the highest AUC of 0.9846. The abu-urban-3 dataset (Table 4) shows that the best performance is obtained with a dual-window size of 21×23, resulting in an AUC of 0.9979. For the Salinas-simulated dataset (Table 5), the optimal dual-window size is 17×19, with an AUC of 0.9993.
  These results indicate that optimal dual-window configurations vary across different datasets, underscoring the importance of dataset-specific parameter tuning. The grid search approach ensures that the selected dual-window sizes maximize the model’s performance, thereby contributing to the overall effectiveness of the proposed method.

**Table 2:** Grid search results of dual-window parameters on AVIRIS-I dataset.

Inner\Outer	
		13	15	17	19	21	23	25	27	29	31	33	35
11	0.9979	0.9983	0.9984	0.9986	0.9987	0.9987	0.9987	0.9985	0.9982	0.9975	0.9970	0.9967
13		0.9983	0.9984	0.9985	0.9987	0.9987	0.9987	0.9986	0.9982	0.9974	0.9969	0.9966
15			0.9985	0.9986	0.9987	0.9988	0.9988	0.9986	0.9982	0.9972	0.9967	0.9964
17				0.9984	0.9986	0.9988	0.9988	0.9986	0.9980	0.9969	0.9964	0.9960
19					0.9985	0.9987	0.9987	0.9984	0.9978	0.9964	0.9958	0.9954
21						0.9989	0.9988	0.9984	0.9976	0.9960	0.9953	0.9949
23							**0.9990**	0.9984	0.9974	0.9955	0.9947	0.9942
25								0.9984	0.9972	0.9950	0.9942	0.9937
27									0.9969	0.9942	0.9934	0.9930
29										0.9923	0.9921	0.9921
31											0.9903	0.9909
33												0.9892

**Table 3:** Grid search results of dual-window parameters on AVIRIS-II dataset.

Inner\Outer												
	13	15	17	19	21	23	25	27	29	31	33	35
11	0.9812	0.9832	0.9839	0.9841	0.9838	0.9834	0.9827	0.9820	0.9815	0.9811	0.9805	0.9803
13		0.9829	0.9841	0.9844	0.9841	0.9837	0.9829	0.9823	0.9818	0.9813	0.9808	0.9807
15			0.9841	0.9845	0.9842	0.9837	0.9829	0.9822	0.9817	0.9813	0.9809	0.9807
17				**0.9846**	0.9841	0.9835	0.9826	0.9819	0.9814	0.9811	0.9807	0.9805
19					0.9834	0.9826	0.9819	0.9812	0.9809	0.9806	0.9802	0.9802
21						0.9819	0.9812	0.9806	0.9803	0.9800	0.9798	0.9799
23							0.9805	0.9798	0.9797	0.9796	0.9794	0.9796
25								0.9796	0.9795	0.9794	0.9793	0.9796
27									0.9800	0.9796	0.9795	0.9797
29										0.9795	0.9793	0.9796
31											0.9795	0.9796
33												0.9805

**Table 4:** Grid search results of dual-window parameters on abu-urban-3 dataset.

Inner\Outer												
	13	15	17	19	21	23	25	27	29	31	33	35
11	0.9952	0.9959	0.9958	0.9905	0.9754	0.9299	0.9049	0.8543	0.8082	0.7768	0.7493	0.7366
13		0.9963	0.9967	0.9935	0.9829	0.9514	0.9171	0.8649	0.8153	0.7824	0.7549	0.7412
15			0.9970	0.9959	0.9857	0.9693	0.9359	0.8793	0.8260	0.7890	0.7639	0.7454
17				0.9974	0.9945	0.9816	0.9603	0.8995	0.8397	0.8009	0.7725	0.7524
19					0.9977	0.9964	0.9830	0.9312	0.8669	0.8178	0.7876	0.7658
21						**0.9979**	0.9903	0.9545	0.8916	0.8413	0.8011	0.7809
23							0.9966	0.9716	0.9074	0.8636	0.8215	0.8031
25								0.9870	0.9265	0.8852	0.8359	0.8155
27									0.9696	0.9236	0.8756	0.8406
29										0.9700	0.9188	0.8755
31											0.9632	0.9207
33												0.9746

**Table 5:** Grid search results of dual-window parameters on Salinas-simulated dataset.

Inner\Outer	
		13	15	17	19	21	23	25	27	29	31	33	35
11	0.999925	0.999925	0.999923	0.999924	0.999919	0.999917	0.999916	0.999919	0.999921	0.999922	0.999920	0.999924
13		0.999928	0.999927	0.999927	0.999925	0.999926	0.999925	0.999925	0.999924	0.999928	0.999927	0.999930
15			0.999928	0.999927	0.999926	0.999928	0.999927	0.999927	0.999925	0.999930	0.999930	0.999931
17				**0.999930**	0.999927	0.999928	0.999930	0.999930	0.999929	0.999931	0.999931	0.999933
19					0.999930	0.999930	0.999929	0.999930	0.999931	0.999933	0.999932	0.999933
21						0.999932	0.999932	0.999932	0.999933	0.999934	0.999933	0.999934
23							0.999933	0.999938	0.999934	0.999933	0.999934	0.999934
25								0.999935	0.999934	0.999935	0.999934	0.999935
27									0.999932	0.999935	0.999936	0.999936
29										0.999932	0.999934	0.999934
31											0.999931	0.999935
33												0.999934

**Fig 10:**
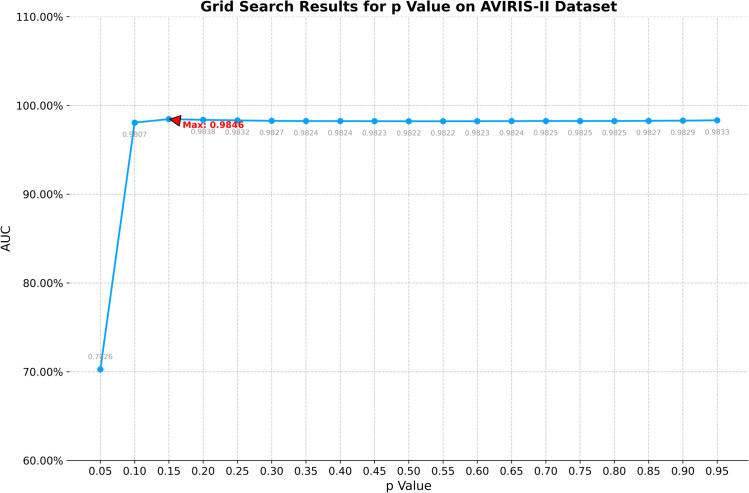
Grid search results for p value on AVIRIS-II dataset.

**Fig 11:**
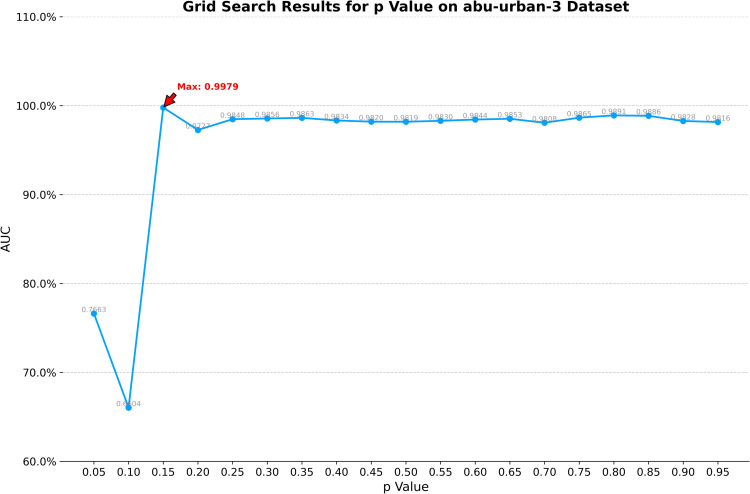
Grid search results for p value on abu-urban-3 dataset.

**Fig 12:**
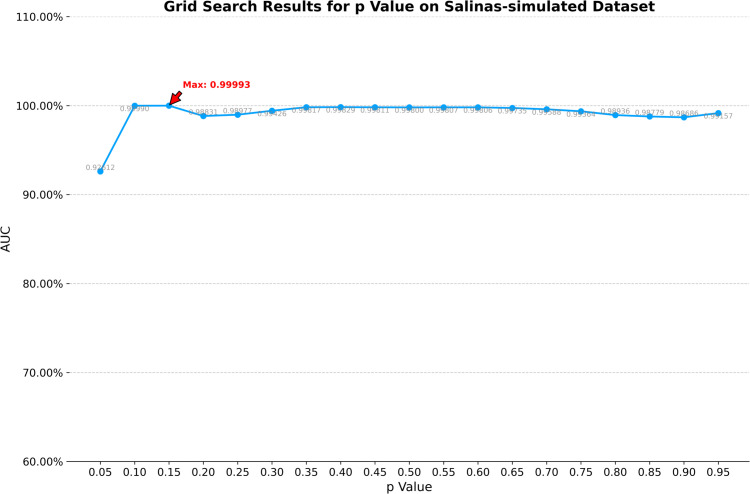
Grid search results for p value on Salinas-simulated dataset.

## Computational complexity

The computational complexity of the proposed ETRPCA-CRD algorithm is a critical aspect for practical implementation. The algorithm consists of two main components: the enhanced tensor robust principal component analysis (ETRPCA) and the collaborative representation detection (CRD).

In ETRPCA, the tensor $𝒴\in {\mathbb{R}}^{n_{1}\times n_{2}\times n_{3}}$ is transformed into the frequency domain via FFT, decomposing the problem into *n*_3_ independent frontal slices. This allows parallel processing of each slice, reducing the complexity from coupled multi-dimensional operations to independent matrix problems. The FFT/IFFT operations exhibit a complexity of: $𝒪(n_{1}n_{2}n_{3}\log n_{3})$. This linear scaling with *n*_3_ (spectral bands) is achieved through the efficient FFT algorithm, which avoids the exponential complexity of direct tensor operations.

T-SVD leverages the block-diagonal structure of the frequency-domain tensor to decompose each frontal slice into orthogonal matrices and a diagonal singular value matrix. For each slice, the SVD complexity is: $𝒪(n_{1}n_{2}^{2})\;(\text{assuming }n_{1}\geq n_{2})$. With *n*_3_ slices processed independently, the total SVD complexity becomes: $𝒪(n_{1}n_{2}^{2}n_{3})$. This avoids the cubic complexity of traditional tensor decompositions by converting the problem into parallel matrix operations.

GST applies adaptive thresholds to singular values, prioritizing large values and suppressing small ones. For each slice, the GST operation involves element-wise soft-thresholding with complexity: $𝒪(n_{1}n_{2})$. Across all slices, this sums to: $𝒪(n_{1}n_{2}n_{3})$ By avoiding uniform regularization of all singular values, GST reduces unnecessary computations and accelerates convergence.

the CRD step, the algorithm involves solving a least squares problem for each pixel in the image. For an image of size $n_{1}\;\times \;n_{2}$, the complexity of solving the least squares problem for each pixel is $O(n_{3}^{2})$, assuming the number of selected background samples is *n*_3_. Therefore, the total complexity of CRD is $O(n_{1}n_{2}n_{3}^{2})$. Combining these, the overall computational complexity of the ETRPCA-CRD algorithm is dominated by the tensor decomposition step, which is $O(n_{1}n_{2}^{2}n_{3}+n_{1}n_{2}n_{3}\log n_{3})$.

Notably, the CRD component in the ETRPCA-CRD algorithm poses a limitation. Its double-window mechanism for solving least squares problems at each pixel results in a high computational complexity of $O(n_{1}n_{2}n_{3}^{2})$. This can slow down processing, especially for high-dimensional data or large images, potentially limiting the algorithm’s performance in time-sensitive or resource-constrained scenarios.

## Conclusions

In conclusion, this paper proposes the ETRPCA-CRD method for hyperspectral anomaly detection, which integrates enhanced tensor robust principal component analysis and collaborative representation to effectively leverage spectral-spatial information. By decomposing hyperspectral data into low-rank and sparse tensors via weighted tensor Schatten-p norm minimization, the approach achieves robust background purification and improved anomaly detection accuracy. Experimental results on multiple datasets validate its superiority over state-of-the-art methods in terms of detection performance and reliability.

Notably, the current implementation involves two areas for potential refinement. The dual-window size in the CRD component and the *p* value in weighted tensor Schatten-p norm minimization are currently determined via rid search to optimize detection results, which may require dataset-specific adjustments . Additionally, while the CRD mechanism ensures precise anomaly identification, its computational complexity—stemming from the dual-window least squares solving—presents opportunities for efficiency improvements, especially in high-dimensional data scenarios. Future research will focus on developing automated parameter selection frameworks to reduce dependency on rid search, such as adaptive algorithms that dynamically tune dual-window sizes and *p* values based on data characteristics. Furthermore, exploring parallel computing architectures and approximate solution strategies for the CRD component will be prioritized to enhance computational efficiency while maintaining detection accuracy, enabling broader applications in real-time hyperspectral monitoring systems.
